# Reading from Single Versus Multiple Displays: A Cross-Sectional Developmental Comparison

**DOI:** 10.3390/brainsci15121284

**Published:** 2025-11-29

**Authors:** Sara Conforti, Marialuisa Martelli, Pierluigi Zoccolotti, Chiara Valeria Marinelli

**Affiliations:** 1 Department of Psychology, Sapienza University of Rome, 00185 Rome, Italy; sara.conforti@uniroma1.it (S.C.); marialuisa.martelli@uniroma1.it (M.M.); or pierluigi.zoccolotti@crtspa.it (P.Z.); 2 Tuscany Rehabilitation Clinic, 52025 Montevarchi, Italy; 3 Cognitive and Affective Neuroscience Lab, University of Foggia, 71122 Foggia, Italy

**Keywords:** reading, multiple stimuli, literacy acquisition, serial superiority effect, serial processing

## Abstract

**Background/Objectives**: Mastery of reading requires the ability to process multiple stimuli in sequence. Previous research shows that children gradually develop this skill as their reading experience increases. This study investigated the serial superiority effect and its association with reading experience. Reading performance was compared using single- and multiple-word displays in typically developing Italian readers from 1st to 3rd grade. Given the link between reading times and interindividual variability, the analyses used models (DEM, RAM, and State trace) that account for global components of individual performance. **Methods**: Ninety 1st-, 2nd-, and 3rd-grade students participated. Children were presented with words of varying lengths. Stimuli appeared either sequentially from left to right (single-display condition) or in a static array of 25 words (multiple-display condition). **Results**: Reading times were faster in the multiple-display than the single-display condition, as expected. Analyses of global components showed that this advantage increased linearly with years of schooling. This finding reflects a progressive mastery of multiple displays as the reading experience grew. **Conclusions**: Global processing models effectively capture early reading acquisition, especially the increasing difference between reading from single displays and managing multiple reading stimuli.

## 1. Introduction

This research investigates differences in reading orthographic stimuli presented in single versus multiple displays among readers in 1st through 3rd grade. Fluent reading requires the integration of various skills, including ocular movements and phonological and orthographic abilities, which depend on both serial and parallel processing of visual information. The motivation for this study arises from the well-documented observation that children with dyslexia exhibit poor performance even on tasks involving serial processing of visual stimuli that do not require reading, such as rapid automatized naming (RAN) [1,2,3,4,5,6,7,8]. While prominent models of reading, including the dual-route cascaded model (DRC) [9], the CDP+ model [10], and the triangle model [11], primarily address reading at the single-word level, difficulties in rapid naming suggest impairments that extend beyond single-word reading and involve the serial processing of multiple stimuli. This is particularly important because everyday reading typically involves meaningful texts. Therefore, exploring how children learn to process multiple stimuli can enhance our understanding of this process and may also shed light on learning difficulties associated with the acquisition of reading skills.

Some studies have looked at how serial and discrete naming relate to reading in children both with and without dyslexia. De Jong [3] studied Dutch children in 1st, 2nd, and 4th grade and found age-related differences. For younger children, serial naming tasks were better at predicting both serial and discrete word reading than discrete naming tasks. For older children, serial naming tasks predicted serial reading, while discrete naming tasks predicted discrete reading. This suggests that serial RAN is linked to within-word reading in younger children and to between-word reading in older children. Protopapas et al. [12] found that, in 2nd-grade Greek children, serial and discrete word reading were strongly related. This means that, at this age, reading words in a sequence is still slow and not much different from reading one word at a time. By 6th grade, serial and discrete tasks were more closely grouped, and the link between serial and discrete reading was much weaker.

Other studies looked at these relationships in groups of children with different reading skills. Altani et al. [13] found that the link between word recognition speed and reading fluency got weaker as children got older, no matter the reading context. They suggested that learning to process groups of words, not just single ones, helps explain how children move from reading word by word to reading whole sequences. Van den Boer and de Jong [14] studied Dutch 2nd, 3rd, and 5th graders using digit, word, and nonword naming tasks. They found some children read letter strings one at a time (serially), while others did so all at once (in parallel). This showed a shift from serial to parallel reading as children develop. Altani et al. [15] showed that serial and discrete reading tasks are similar at first, but their link weakens as children get older and better at reading. At the same time, the connection between serial naming and serial reading gets stronger. This means that reading lists of words becomes more like naming a series of familiar items. Recently, van de Boer et al. [16] followed 181 children from 2nd to 4th grade. They did serial and discrete RAN, and word and pseudoword reading tasks. The link between serial and discrete reading dropped sharply between 3rd and 4th grade. The connection between serial RAN and serial reading grew stronger between 2nd and 3rd grade. These results show that, as children grow, they get better at reading several words at once.

Evidence in the studies mentioned above predominantly relied on a correlational approach; however, additional research focused on comparing reading speeds for stimuli presented in multiple displays versus those presented as discrete items. These studies revealed that children who follow the expected trajectory of literacy learning (hereafter, typically developing children) tend to read faster with multiple displays than with discrete items, a phenomenon known as the “serial superiority effect” [8,12,17,18]. In their research, Altani et al. [19] found the serial superiority effect across various tasks (digits, objects, dice, number words, and words) among 720 English- and Greek-speaking children from 1st, 3rd, and 5th grade. The improvement in the serial advantage between grades was largest for word reading, while it initially resembled object naming in 1st grade. The coordination of multiple elements and processes, both in parallel and serially, has been termed “cascaded processing” [12]. Additionally, the term “between-word processing” has been used to refer to the skills required for the simultaneous processing of multiple words [20]. Notably, this process was found to be critical even for nonlinear/non-alphabetic orthographies, such as Korean and Chinese [21].

Efficiently processing several words in a row helps explain the link between serial naming and serial reading [12,22]. Serial digit naming can show how well someone processes sequences, since naming familiar digits lets people quickly match symbols to sounds, much like reading words by sight [22]. Processing words or symbols in a sequence can happen both at the same time and one after another, so the next item may start being processed before the previous one is finished. This overlap leads to the serial superiority effect seen in word reading, where people read word lists faster than single words, especially for more experienced readers [22]. Romero et al. [23] found that for 5th-grade English-speaking children, whose word recognition was likely automatic, serial naming explained differences in how quickly they could read word lists when comprehension was not a major factor. Serial naming also predicted growth in serial reading from 2nd to 5th grade. Similarly, van Viersen and colleagues [20] studied 139 Dutch students in 3rd and 5th grades and found that processing between words is important for fluent reading, whether the words are simple or complex. This was true for both intermediate and advanced readers, and the role of between-word processing grew as reading skills improved.

A key challenge in comparing single and serial naming tasks is the use of qualitatively different performance measures. In single naming, researchers typically measure the time from when the stimulus appears to when the participant begins their response. This is known as reaction time (RT), and it is widely recognized in the literature as it is intended as a sensitive indicator of the ability to decode the stimulus. In contrast, pronunciation times are generally not considered significant, as they show minimal individual differences and have little impact on distinguishing groups, such as children with dyslexia and their chronologically matched children [24]. When assessing texts or lists of words, reading time encompasses both the reaction time and the duration required to articulate the sentences or words. This combined measure is commonly employed in clinical practice to evaluate reading disorders in children with dyslexia. Consequently, it is necessary to assess total reading times, including both RT and pronunciation time, for singly presented words to enable valid comparisons of reading fluency between discrete and multiple displays [18]. Thus, examining the acquisition of reading skills requires experimental conditions that facilitate direct comparison of performance with single and multiple displays.

The present investigation aimed to study the serial superiority effect as a function of reading experience in the early stages of literacy acquisition. To investigate the relationships between single and multiple displays, we sought to overcome some possible methodological criticisms. First, we devised a paradigm that would allow a direct comparison of single and multiple conditions. In the multiple-display condition, we presented an array of unrelated words simultaneously. In contrast, the single condition involved presenting words one at a time from left to right, line by line. To minimize any potential confounding factors related to the location and appearance of the target word, we included sound and visual markers in the single display condition. This approach ensured that visual attention was captured in a bottom-up manner by a salient external trigger [25]. In the multiple-display condition, there was no external trigger; therefore, readers had to rely on their own internal pacing to read the stimuli fluently. This distinction may lead to greater differences between groups in the multiple-display condition compared to the single display condition [18].

In previous research involving children with and without reading deficits, we found that reading times for both multiple-word and single-word displays exhibited significant co-variation between performance and individual variability. This relationship challenges the assumption of homogeneity of variance. In such cases, traditional parametric analyses may overestimate group differences under more difficult conditions, with the slower group showing a greater effect than the faster group. This phenomenon is commonly referred to as the over-additivity effect [26]. In contrast, several authors have proposed alternative models, such as the Difference Engine Model (DEM) [27], the Rate and Amount Model (RAM) [26], and State trace analysis [28], which focus on identifying global—non-task-specific—components in the experimental data, controlling for over-additivity effects. While these models approach the issue from different angles, they provide complementary insights into global components.

Both RAM and DEM seek to measure individual differences in performance during timed tasks, particularly reaction times (RTs), although they do so from slightly different perspectives. According to RAM [26], performance on any task is influenced by two factors: the difficulty of the task (or the “amount” of information that needs to be processed) and the individual’s basic speed of processing (the “rate”). These two factors interact multiplicatively, meaning that the difference between a slower and a faster group will be more pronounced on difficult tasks compared to simpler ones, resulting in the over-additivity effect. The DEM [27] aims to describe the global factor by isolating two general components in responses, known as compartments. One compartment is associated with sensory-motor components, while the other pertains to decisional aspects. These compartments can be distinguished based on the relationship between the means of conditions and their corresponding standard deviations (SD). State trace analysis [28] is another approach that addresses individual performance by considering global influences within the data. Unlike RAM and DEM, which primarily focus on RTs, state trace analysis can also apply to accuracy measures (often generating nonlinear relationships), although it may also extend to the RT paradigm (e.g., [29]). This method examines whether the relationship between a set of causal variables and a set of indicator variables is mediated by one or more latent variables. In this context, the factor associated with the indicator variables is referred to as the state factor (in the present study, this is the display size), while the dimension factor (in this study, school grade) influences the mediating latent process. Consequently, tests based on these models, as detailed in the Data Analysis section, allow for a comprehensive evaluation of how global components affect reading times, taking into account both group differences related to reading experience and display types. This approach controls for various influences, including both over-additive and under-additive effects, where slower individuals may produce smaller effects. This method is akin to approaches previously utilized in aging research [29].

In this cross-sectional developmental study, we aimed to compare a reading task that involved both single-word and multiple-word displays among typically developing readers from 1st to 3rd grade (i.e., an early stage of reading development). Our objective was to confirm the advantage in reading words from multiple over single displays (serial superiority effect) and observe how it evolves during the initial phases of literacy acquisition. Based on previous research, we expected the serial superiority effect to increase as a function of reading experience beyond overall changes in performance. To this goal, in our analyses, we referenced several models, including DEM, RAM, and State trace, which consider the overall components of individual performance.

## 2. Materials and Methods

### 2.1. Participants

Participants included 90 typically developing readers (45 males and 45 females): 30 children in 1st grade, 30 in 2nd grade, and 30 in 3rd grade. Table 1 presents the sociodemographic characteristics of the samples, as well as their reading and reasoning performance. All children scored within normal limits on cognitive assessments (not below the 5th percentile according to the reference sample at the Colored Progressive Matrices, CPM, of Raven [30]) and reading evaluations (not more than one standard deviation below the mean of the reference sample for reading speed and accuracy; details provided below). All children meeting these conditions from the classes involved in the project participated in the study. Children who did not meet the aforementioned criteria for typically developing readers were excluded. We also excluded bilingual consecutive children and children with a positive anamnesis for any other neurodevelopmental disorders in comorbidity. However, we did not carry out a screening for other cognitive factors, such as attention, language, or other learning disorders apart from dyslexia.

We recruited the children from schools with an average/normal socio-economic background in the provinces of Foggia and Rome. All children also had adequate literacy exposure and learning opportunities. Parents provided written informed consent for their children’s participation. The research was reviewed and approved by the Ethics Committee of the University of Foggia, Italy (prof. n° 011/CEpsi of 2 May 2023). All methods were carried out in accordance with the ethical standards of the Declaration of Helsinki.

### 2.2. Reading Assessment

In the MT Reading Test [31], the child must read a passage as quickly and accurately as possible, within a 4-min limit. Reading time (syllable/s) and accuracy (number of errors, adjusted for text read) are scored [30]. Table 1 presents data, both in absolute and normalized z-values (limited to reading times). The sample’s reading performance was within expected norms for both accuracy [30] and reading time, as shown by mean z-scores near zero [31].

Additionally, we used a test requiring the reading of lists of words, the One Minute Reading Test (OMrT—an adaptation of the One Minute Reading Test [32]; see also [18]). One hundred fifty-eight low-frequency words were extracted from the LEXVAR 2 database [33]. Words were all bi-syllabic and 5-letter long, with a frequency of occurrence lower than 30 (mean = 15.54, SD = 6.44; range 6–30) according to the Children’s Word Frequency database [34]. Due to the small number of letters, selected words had a large neighborhood (mean 8.45, SD = 4.12) according to the Colfis database [35]. The words were presented simultaneously on a page (in a grid format). Children had to read the words aloud as quickly as possible, proceeding from left to right across the page. When they finished a line, they had to go down to the next one. They were stopped after a minute. The score was the number of words read correctly in one min. The OMrT data in Table 1 indicate the substantial increase in word reading from 1st to 3rd grade.

### 2.3. Experimental Task

The stimuli were selected from the LEXVAR 2 database [33]. For both the serial and discrete tasks, the conditions comprised four stimulus blocks. The four blocks consisted of 25 words, one for each of the letter lengths used (4-, 5-, 6-, and 7-letter words). There were two parallel lists, one for the serial condition and the other for the discrete condition. Parallel lists did not differ on any psycholinguistic variables examined. The words had a mean frequency of 58.83 (SD = 82) according to the children’s word frequency count [34]. Subsets of words did not differ from each other for the age of acquisition, familiarity, imageability, children’s word and bigram frequency [34], contextual rules, letter confusability, and point of articulation of the first phoneme. As expected, the lists of words of different lengths differed between them for variables co-varying with length, such as number of phonemes, ortho-syllabic complexity (number of consonant clusters and geminate consonants), and neighborhood size (N-size, i.e., the number of words differing by only one letter to a target word).

**Figure 1:**
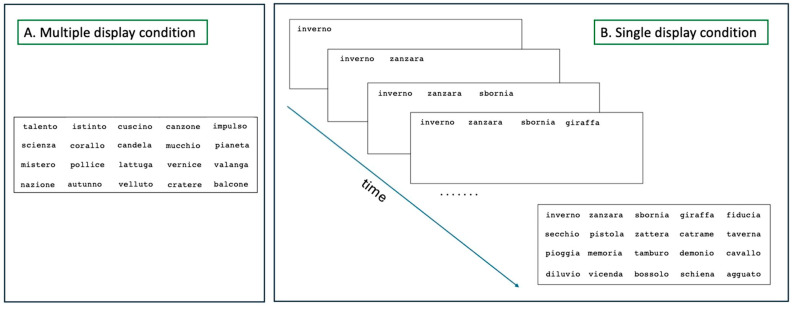
(**A**) Example of a trial in the multiple-display condition. (**B**) Example of a trial in the single display condition: the words appeared one by one (remaining on the screen). The example illustrates the progressive appearance of the first four words and, subsequently, the final display (with 20 target words).

There were two conditions with four experimental blocks in each one:

- Multiple-display condition: an array of 25 unrelated words appeared simultaneously on a laptop screen (arranged as in Figure 1A). Children had to read aloud all words as quickly as possible, from left to right and from top to bottom. Words remained on the screen until the reading of the last word.
- Single-display condition: 25 words appeared one at a time, from left to right, marked by a sound (“beep”). Children had to read the words aloud as they appeared as quickly as possible. Every word appeared after 3 s from the onset of the previous child’s response. Once the words appeared, they remained on the screen (see Figure 1B).

Words appeared in black lowercase Courier font (characterized by a constant centre-to- centre letter spacing) on a white background. Participants were tested individually in a quiet room at their school. They were seated 57 cm away from the screen, and the x-width measured 1.06 deg. For both multiple and single displays, item presentation and response recording were controlled by the MATLAB & Simulink R2015b experimental software.

Total reading times for items responded to correctly were considered for the analyses. In each condition, we counted the number of reading errors. Utterances that did not correspond to the presented word, self-corrections, stress errors, as well as word omissions were considered errors. Individual responses were recorded in audio files through an external computer microphone. For single and multiple tasks for each condition (5-letter word, 6-letter word, etc.), we recorded a single audio track.

Single and multiple tasks. For both the multiple- and single-display conditions, total reading times were determined offline using an audio editor and recorder (Audacity 2.1.3 Version). Thus, our dependent variable was the total reading time for correct responses only: mean total reading times [RTs + pronunciation time] for the single-display condition and [pause + pronunciation time] for the multiple-display conditions.

For the single-display condition, the total reading time of all items was considered, including both onset latency and pronunciation time. We eliminated the pauses between the end of the pronunciation and the subsequent “beep” as well as the errors. If the child did not read the word within 3 s before the next word appeared, we deleted the reading time of the unread word and that of the next word. Sometimes, reading the two words overlapped in the same window of time (i.e., the child read the word after the next appearance), creating a situation of multiple reading. In these cases, we did not consider either time. However, this occurred quite rarely (less than 2% of cases). The total reading time (in seconds) of each condition (4-letter words, 5-letter words, 6-letter words, and 7-letter words) divided by the number of words read correctly yielded an estimate of “s per item”. For the multiple-display conditions, we considered the total reading time of all items, including pauses and pronunciation times, but eliminated the erroneous pronunciations from the total time. The total reading time of each condition divided by the number of words read correctly yielded an estimate of “s per item”.

The order of single and multiple-display conditions was counter-balanced among participants. Each experimental session lasted approximately 15 to 20 min, with a short break between the single and multiple condition tasks to prevent participant fatigue. A brief practice session was conducted before each experimental session to ensure that the children understood the instructions clearly. Participants were tested individually in a quiet room at their school.

### 2.4. Data Analysis

We explored the differences between children in various grades from a global perspective, i.e., a non-task-specific approach.

First, we tested the prediction of a linear relationship between the overall group means of total reading times and the standard deviations (SDs) under the same conditions [27], i.e., that more difficult conditions are associated with larger inter-individual differences in performance. According to Myerson et al. [27], this type of plot identifies the basic relationship underlying global components in the data (see also [36]); i.e., the model predicts that the condition means of different groups of subjects varying for overall speed of processing (e.g., older, and younger adults) will lie on the same quasi-linear regression (even though on different part of the line). According to DEM, the intercept on the x-axis marks the part of the response devoted to the perceptual identification and motor programming of the response (“sensory–motor compartment”); the model hypothesizes that this part of the response is independent of the task difficulty (as assessed by experimental condition means) and is relatively invariant even in groups varying for overall speed processing (such as older adults compared to young adults). In contrast, the slope of the linear relationship marks the “cognitive compartment”, which is sensitive to task difficulty (manipulated here through word length), as well as to differences in global processing speed.

It is important to note that the DEM focuses on reaction times (RTs) rather than reading times (which also include pronunciation times). Previous research on children with and without a reading difficulty indicated that pronunciation times vary minimally as a function of the reading difficulty [24]. However, pronunciation times do not merely add a constant value as they may vary as a function of condition, such as word length [24]. Therefore, this fact makes the expectation somewhat more complex. In the case of reading times, the regression slopes for children with dyslexia and controls differed, as pronunciation times weighted more heavily onto the responses of control children than those of children with dyslexia [24]. However, for a cross-sectional developmental analysis, as the one envisaged here, one may expect that both RTs and pronunciation times vary as a function of age/reading experience. If so, one may expect data of children of different ages to lie on the same regression line, even for reading times. Note that we did not record separate measures for pronunciation times. Therefore, we could test this prediction only indirectly based on the present data.

Second, to examine global differences between grade groups, we investigated the linear relationship between the condition means of children in higher and lower grades (i.e., 2nd vs. 1st and 3rd vs. 2nd) using Brinley plots. This analysis helps identify group differences controlling for the over-additivity effect, i.e., the tendency for larger group differences in more difficult conditions of a “slower” group [26]. The slope of the linear relationship indicates the extent of the global change in performance after one year of literacy instruction. The Brinley plot analysis is typically applied to RT measures; however, previous research has shown that it may also be effective with reading times [18,24]. As reading times include pronunciation (which is expected not to vary much across groups with different global processing speeds), the slope of the Brinley plot is expected to be somewhat attenuated for reading times compared to RTs [36].

Finally, we referred to the State trace analysis [28] to study the relationship between single and multiple presentations at different stages of reading development (from 1st to 3rd grade). Note that, while the use of DEM and RAM is restricted to open scales (and, hence, time measures), the State trace analysis is a general form of analysis that may be applied independently of the type of scale, and, indeed, it has already been used with reading time measures [37]. The means of the multiple-display conditions are plotted against the means of the single-display conditions. In this analysis, the dimension factor is grade (1st, 2nd, and 3rd), and display size (single vs. multiple conditions) is the state factor. The goal is to examine whether the relationship between the causal and indicator variables is mediated by one or more latent variables.

## 3. Results

**Figure 2:**
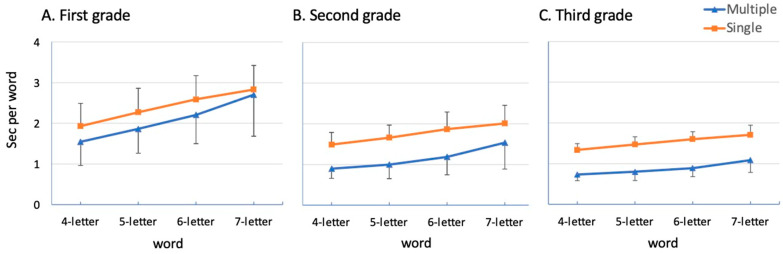
Mean total reading times (s for each word) for typically developing readers attending 1st, 2nd and 3rd grade.

Figure 2 presents the mean reading times (time per item, expressed in s) for correctly read words (out of a total of 25 words) in the single and multiple-display conditions for children in 1st to 3rd grade. Inspection of Figure 2 indicates that reading times were progressively slower with increasing word length, a tendency more evident in the 1st grade. Children in 2nd and 3rd grade exhibited faster reading times when reading words in multiple displays than in single displays. The children in 1st grade showed a less marked advantage for multiple displays and no advantage for long words.

### 3.1. Condition Means Versus Standard Deviations

**Figure 3:**
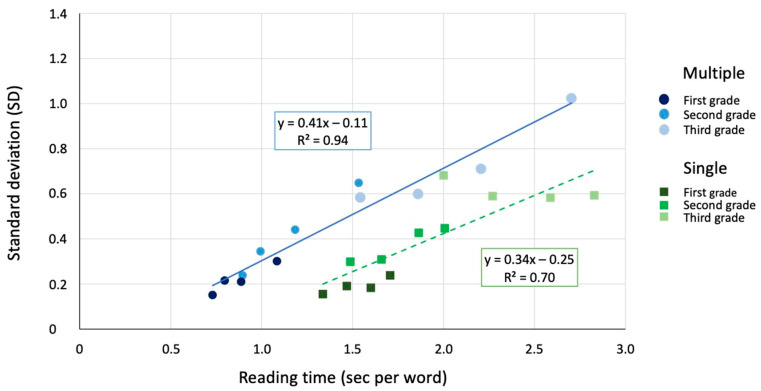
SDs of children attending 1st, 2nd and 3rd grade are plotted as a function of multiple (circles) and single (squares) conditions means in total reading times. Two regression lines, one for the multiple- and one for the single-display condition, fit the data best.

To examine the presence of global components in the data, we first examined the relationship between the SDs and the condition means in total reading times [26] (see Figure 3). An inspection of this figure indicates various main observations:

− SDs (i.e., inter-individual variability) grow linearly as a function of the mean reading times, indicating systematic violations from the assumption of homogeneity of variance and the presence of global components in the data.
− There is a larger spread of performances in the case of multiple-display conditions than in the case of single-display conditions.
− Two separate linear regressions (one for the single- and one for the multiple-display conditions; see Figure 3) best explain the data; using a single regression line yielded a lower coefficient of determination (y = 0.28x − 0.04; R^2^ = 0.56).
− The data of children in different grades lie on the same regression line, as typically observed in the case of RT measures.
− The fit for the multiple-display conditions is very high (R^2^ = 0.94), while that for the single-display conditions is somewhat lower (R^2^ = 0.70). Inspection of the figure indicates that children in 1st grade showed a somewhat deviant pattern with high SDs, independent of condition manipulation (i.e., stimulus length).
− Data for the multiple-display conditions are shifted to the left, as indicated by a smaller intercept on the x-axis (0.268 s) with respect to single-display conditions (0.735 s); by contrast, the size of the slope of the two regression lines is similar.

### 3.2. Brinley Plot Analysis

**Figure 4:**
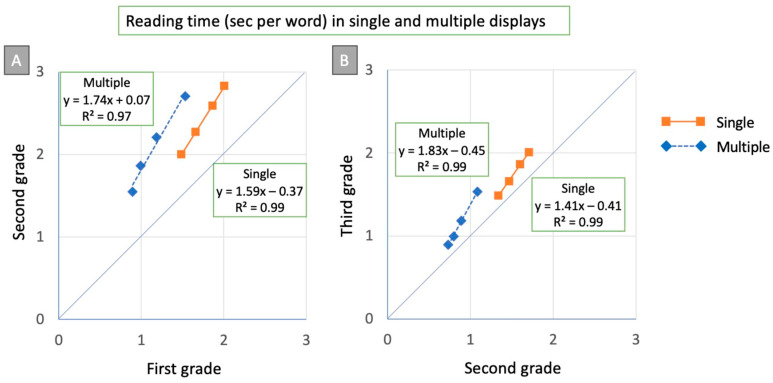
Brinley plot analysis: (**A**) The condition means of children in 2nd grade are plotted against the respective condition means of the children in 1st grade, separately for the single display conditions (ochre squares) and the multiple-display conditions (blue diamonds). Two different regression lines (one for the multiple- and one for the single-display conditions) fit the experimental data well. The diagonal line in the plot represents the equality line, indicating the absence of group differences. (**B**) The condition means of children in 3rd grade are plotted against the respective condition means of children in 2nd grade. Two different regression lines (one for the multiple and one for the single display conditions) fit the experimental data well.

Next, we examined reading times as a function of reading experience by Brinley plot analysis [26] (see Figure 4). Inspection of plot A in the figure indicates that performance improvement from first to second grade is well described by two regression lines, one for the single- (R^2^ = 0.99) and one for the multiple-display (R^2^ = 0.97) conditions. The two regression lines have similar slopes (i.e., 1.59 and 1.74, respectively) but different intercepts on the ordinate axis. Similar results emerge from plot B of the figure, which shows the changes in performance from 2nd to 3rd grade. Two regression lines (one for the single- and one for the multiple-display conditions; both R^2^ = 0.99) fit the data well. However, note that in this case, the two regression lines have similar intercepts, while the regression for the multiple-display condition tends to have a somewhat higher slope (1.83 vs. 1.41).

### 3.3. State Trace Analysis

**Figure 5:**
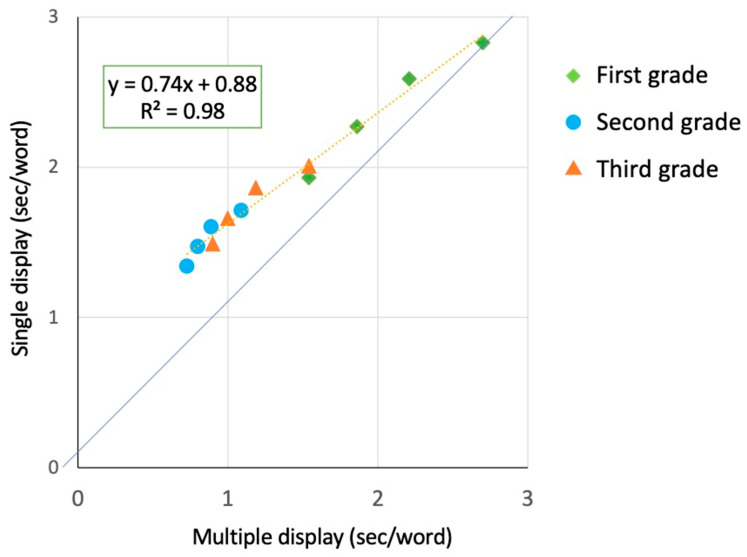
State trace analysis: single-display condition means of children in 1st, 2nd, and 3rd grade are plotted against the multiple condition means of the same children. One regression line fits all data points (with R^2^ = 0.98 and beta = 0.74).

Figure 5 presents data as state trace plots [28]; i.e., the mean reading times in the multiple-display conditions for children in 1st, 2nd, and 3rd grade are plotted as a function of the means of the corresponding single-display conditions. In the figure, the diagonal line represents the equality line, i.e., no difference between the conditions. Experimental points for all three groups of children are explained well by a single regression line (R^2^ = 0.98). Data for children attending 1st grade are close to the diagonal line. The distance of experimental points from the equality line progressively increases from the 1st (green diamonds) to the 2nd (ochre triangles) to the 3rd (light blue circles) grade. Thus, the difference between single and multiple conditions increases with reading experience (and faster reading times). Therefore, this effect indicates an under-additive tendency, such that greater differences between the critical conditions are associated with faster reading times and consequently quantitatively smaller differences in absolute reading times. Overall, one observes a progressive emergence of a reading advantage for multiple-display conditions across the grades considered.

## 4. Discussion

To capture global components, it is crucial to obtain a sufficiently wide range of performance across the experimental conditions. Accordingly, task difficulty was varied by manipulating word length. As expected, children tended to read longer words more slowly than shorter words. Based on previous studies, it is likely that the influence of length in the early phases of reading acquisition depends upon both the decoding [38] and the articulation [36] components of the response. Note that, in the present study, we did not separately analyze these two components. Therefore, the present data do not allow for evaluating the relative weight of these two components. When examined by matching condition means against inter-individual variability, a single regression line accounted well for the performance across conditions (i.e., word lengths) of single-display stimuli. This outcome is predicted by the DEM [26] and is typical of studies examining RT data [36]. As regards reading times, the prediction is somewhat more complex. Martelli et al. [24] reported different regression slopes for children with dyslexia and control readers (similar results are reported in [18]). This finding may be due to a dissociation between the various components of the response, characterized by quite different levels of interindividual variability [24]. Thus, in children with dyslexia, word length influences both decoding and articulation times; in contrast, in control readers, word length influence is almost entirely limited to the pronunciation components. The present observation that reading times in typically developing children have a single relationship with interindividual variability may indicate that the decoding and pronunciation components in reading develop harmoniously in these children.

The finding of a single regression line for reading times in single-word reading is broadly consistent with the idea from previous research [38,39] that, by and large, performance in reading single words may be accounted for based on a single global factor of orthographic processing. This factor provides the pre-lexical “grapheme description”, independent of case, font, location, or orientation, which represents the base for all subsequent forms of processing (including reference to the lexicon) [40]. Even for multiple-display stimuli, reading performance was well accounted for across different word lengths by a single regression line. The two regression lines differed in their intercepts, i.e., the data for the multiple-display conditions were shifted to the left by approximately 500 ms. This finding indicates an advantage in reading from multiple over single displays, i.e., the serial superiority effect [8,12,17,18]. These data are consistent with several previous observations [8,12,17] and indicate that children can analyze the next stimulus before the complete processing and production of the previous one, resulting in partial temporal overlap [8,22].

The Brinley plot and State trace analyses indicated a progressive emergence of a reading advantage for the multiple-display conditions in the grades considered. Furthermore, both types of analyses clearly confirmed the presence of global components in the data. Thus, in the Brinley plot analysis, the shift in reading performance from 1st to 2nd grade was well described by a single regression line, and the same held for the change between 2nd and 3rd grade. Again, the performance for the single- and multiple-display conditions was clearly differentiated. Notably, the increase in performance in the multiple-display condition (as indicated by the slope of the regression) was similar when passing from 1st to 2nd grade and from 2nd to 3rd grade. Therefore, the entire period under consideration seems critical for acquiring the ability to process multiple orthographic stimuli. In future research, it would be interesting to examine further stages of development in reading acquisition.

Furthermore, the State trace analysis clearly documented the progressive emergence of the serial superiority effect. In 1st grade, the serial superiority effect was comparatively small; however, the advantage increased linearly with schooling up to 3rd grade, indicating a progressive mastery of multiple displays with increasing reading experience. Note that the differences between single- and multiple-word displays, i.e., the serial superiority effect, increased with decreasing reading times, i.e., when children become faster. This effect is thus under-additive, indicating that larger group differences are associated with shorter reading times, and, consequently, with numerically smaller differences in raw values. Since time is an open-ended scale, it is unlikely that this effect can be interpreted as a ceiling effect. Instead, the under-additivity appears to be related to the general principle that the magnitude of effects is not independent of the individual’s baseline processing level, as proposed in the RAM model [26]. Therefore, one intriguing finding of the study is that an under-additivity effect masks developmental differences between single- and multiple-display conditions, which may be appreciated only partially in the raw data.

One final consideration concerns the idea proposed by some authors (e.g., [18]) that the multiple-display condition may amplify the impact of the manipulated factors (i.e., word length in the present study). Some indications come along this line. Thus, the spread of performances appears larger in the multiple-display conditions; this effect is clearly apparent in Figure 3. As noted above, the presence of an under-additive tendency in the data may partially mask this effect when examining the raw data.

Overall, taking into account global components in processing has allowed us to effectively describe variations in performance between single and multiple displays, confirming previous data on children with dyslexia [18]. This approach effectively captured the early stages of reading acquisition, particularly the gradual transition from reading single displays to mastering multiple reading stimuli. As noted in the introduction, most existing models of reading (such as the dual-route cascaded model (DRC) [9], the CDP+ model [10], and the triangle model [11]) primarily focus on describing the core competence of reading. However, they do not explain how reading occurs in realistic, ecological situations. These models were often developed based on specific deficits observed in patients with localized brain lesions. However, when it comes to describing behavioral deficits in children, the situation is more complex. Instead of experiencing highly selective deficits, children typically display a combination of associated impairments [41,42]. Thus, considering only core competence deficits may fail to provide a comprehensive description of the cognitive impairment in a given child [42,43].

To address this, we have proposed a “multi-level model of learning cognitive skills” [44] that explicitly formulates not just the competencies involved in reading, spelling, and math, but also various “performance” factors, using this term in the sense originally proposed by Chomsky [45], i.e., the actual, concrete use of a cognitive process (language in the case of Chomsky) in real-life situations. Performance is contrasted with “competence”, referred to as the abstract, general capacity to process in a given cognitive domain (such as language) [45]. In explaining fluency in complex tasks, we have proposed that a dimension of “integration of task sub-components” provides a basis for modelling the fluency in performing a range of complex behavioral tasks, such as reading and other learning skills. We further proposed that sensitive measures capturing this dimension are the classical RAN tasks [1,2,3,4,5,6,7,8]. A key piece of evidence supporting this idea is that RAN tasks can predict reading performance when stimuli are presented in a typical serial format. On the contrary, this predictive ability disappears when stimuli appear in a discrete, single-instance format (e.g., [5]). Critically, factors contributing to performance components should demonstrate format specificity. Research has consistently shown that the ability to perform well on RAN tasks predicts fluency in reading and math but not accuracy (e.g., [46]). Notably, these relationships have been supported by longitudinal investigations, confirming their unidirectionality [47,48]. Conversely, RAN tasks do not predict performance in spelling, an orthographic task that does not place stringent time constraints for effective performance [49,50].

Another line of research that is potentially relevant to the description of reading acquisition derives from the study of eye movements [51]. It is well-known that recording eye movements allows for a particularly sensitive online description of the reading process. Thus, studies examining eye movements during reading aloud have documented, for a long time, the presence of an asymmetry between visual exploration/recognition and articulation of the text being read (or eye-voice span) [52,53,54]. Furthermore, several models have been developed, such as the E–Z model [55], SWIFT [56], and the OB1-reader [57], which, in different ways, attempt to integrate saccadic programming processes with reading decoding processes. Notably, while the behavioral studies stemming from the RAN literature (described in Section 1) as well as the present one, focus on the examination of individual differences in the ability to manage multiple stimuli in reading, research on eye movements may be instrumental in uncovering the mechanisms that allow the processing of multiple stimuli. However, despite the potential of eye movement research to provide valuable insights into the transition from processing single words to cohesively analyzing multiple targets, there are relatively few studies that describe the early stages of reading acquisition in the context of eye movement models (e.g., [58]). Therefore, this remains an interesting topic for future research.

The observation that the ability to manage multiple stimuli significantly improves during the early years of reading acquisition may have implications for assessing reading difficulties, as well as for educational and rehabilitation practices. For example, in Italy, it is possible to diagnose dyslexia from the third year of primary school onwards. At this stage, children are expected to master sequences of words, rather than simply demonstrating proficiency in reading single words. Therefore, it would be valuable to develop clinical tools that enable the separate evaluation of reading stimuli in single versus multiple formats. In the absence of such a test, it is possible to consider a performance in RAN tests below normative values as indicative of a difficulty in managing multiple stimuli. Furthermore, it might be interesting to develop reading programs that specifically train children to deal with progressively longer word sequences.

### Limitations and Future Perspectives

There are limitations of the present study that should be emphasized. Our only manipulation involved word length. It would be valuable to explore additional psycholinguistic factors, such as word frequency or neighborhood size (N-size). In previous research, we proposed that the global orthographic factor influencing reading performance is prelexical [38,39]. Therefore, it is reasonable to expect that this factor may also account for the reading performance across stimuli that vary in word frequency and N-size. Some evidence supports this idea in the context of single-word reading (e.g., [59]). However, these factors have not been tested with words presented in multiple displays, as done here. Examining the impact of various psycholinguistic factors may help evaluate the idea that reading from multiple displays intensifies the effect of these manipulations. Further research is needed to address this question.

Additionally, potential cohort effects—such as differences in schooling between regions and variations in teaching methods across classes—should be considered. Conducting a replication of this study with a larger sample and a longitudinal design could help control for these potentially confounding factors. Furthermore, it would be interesting to explore how the serial superior effect develops in more experienced (older) readers.

The present study was limited to behavioral measures. Using multiple methodologies, such as EEG, evoked potentials, and eye-tracking, to examine single versus multiple word reading can provide valuable insights into the reading process. As stated above, it may prove particularly interesting in comprehending the mechanisms associated with the processing of multiple stimuli.

The current study examined Italian orthography, which is known for its high consistency. Previous research has identified various cross-linguistic differences in the reading process, as influenced by orthographic consistency. These differences have been linked primarily to the greater reliance on lexical processing during single-word reading in inconsistent orthographies (such as English) compared to more consistent orthographies (e.g., [60]). However, the serial superiority effect has received less attention in cross-linguistic research. A study by Altani et al. [19] demonstrated this effect in children who speak English and Greek, indicating that the serial superiority effect occurs in both consistent and inconsistent orthographies. Nonetheless, further research seems needed to further explore cross-linguistic differences in reading multiple displays.

## 5. Conclusions

The study enhances our understanding of reading decoding in the context of multiple displays, which more closely resembles real-life situations. The findings indicate that models of global processing effectively capture the early stages of reading acquisition, particularly the gradual transition from reading single displays to mastering multiple reading stimuli. Most existing models of reading focus on single-word reading and do not adequately address the progressive development of skills required to handle multiple stimuli that characterize literacy acquisition. Therefore, it seems crucial in developing models of reading to explicitly consider the factors influencing the ability to navigate multiple displays during reading.

## Figures and Tables

**Table 1:** Summary statistics for the groups of participants: mean age in years (with standard deviations, SD, in parentheses); N = number of female and male participants; mean raw scores (and SDs in parentheses) and percentiles on Raven’s Colored Matrices [29]; mean raw scores (and SDs) on reading time and accuracy measures of the MT Reading test [30]; words per min at the OMrT test [31].

Grade	Age	N	Females	Males	Raven Test	Raven Test (Percentile)	Reading Time (sill/s)	Z-Score Reading Time (sill/s)	Reading Accuracy	OMrT
1°	6.9 (0.32)	30	15	15	23 (4.07)	68 (22.03)	1.6 (0.43)	0.2 (0.5)	3.9 (1.6)	32 (9.7)
2°	7.8 (0.34)	30	15	15	26.7 (5.43)	69 (25.7)	2.7 (0.70)	0.7 (0.86)	3.3 (2.1)	55 (16.2)
3°	8.8 (0.46)	30	15	15	28.1 (3.84)	61.3 (25.6)	3.2 (0.55)	0.2 (0.5)	4.5 (2.5)	68.6 (16.06)

## Data Availability

Data will be made available only upon request for reasons of privacy.
